# A Neglected Issue in Ulcerative Colitis: Mesenteric Lymph Nodes

**DOI:** 10.3390/jcm7060142

**Published:** 2018-06-08

**Authors:** Abdurrahman Sahin, Hakan Artas, Yesim Eroglu, Nurettin Tunc, Gulcan Oguz, Ulvi Demirel, Orhan Kursat Poyrazoglu, Mehmet Yalniz, Ibrahim Halil Bahcecioglu

**Affiliations:** 1 Division of Gastroenterology, Department of Internal Medicine, Firat University School of Medicine, 23119 Elazig, Turkey; nurettin@firat.edu.tr (N.T.); drudemirel@hotmail.com (U.D.); okpoyrazoglu@yahoo.com (O.K.P.); mehmetyalniz@hotmail.com (M.Y.); ihbahcecioglu@yahoo.com (I.H.B.); 2 Department of Radiology, Firat University School of Medicine, 23119 Elazig, Turkey; hakanartas@yahoo.com (H.A.); dryesimeroglu@gmail.com (Y.E.); 3 Department of Internal Medicine, Firat University School of Medicine, 23119 Elazig, Turkey; gulcan_2395@hotmail.com

**Keywords:** ulcerative colitis, mesenteric lymph node, attenuation, dimension

## Abstract

Data evaluating the presence and characteristics of mesenteric lymph nodes (LNs) in patients with ulcerative colitis (UC) are scarce. The aim of this study is to determine the presence and characteristics of LNs in UC. The LN characteristics in computed tomography (CT), including LN dimension and attenuation, were evaluated retrospectively in 100 patients with UC (61 active and 39 inactive cases). Clinical characteristics and laboratory parameters, including CBC, biochemical analysis, erythrocyte sedimentation rate (ESR), and C reactive protein (CRP) were also compared. Mesenteric LNs were evident in all patients with UC. The attenuation and dimension of mesenteric LNs did not differ between active and inactive patients with UC. No correlation was found among patients with UC in terms of LN dimension, attenuation, ESR, CRP, leucocyte, and albumin (all with *p* > 0.05). The current study suggested that inflammation results in the development of mesenteric LN in UC, similar to Crohn’s disease and other inflammatory disorders.

## 1. Introduction

Inflammatory bowel diseases (IBDs) are chronic idiopathic inflammatory disorders of the intestine or colon, characterized by chronic inflammation due to unbalanced activation of the mucosal immune system in response to luminal antigens in genetically predisposed individuals, and are mainly classified into two major forms: Crohn’s disease (CD) and ulcerative colitis (UC). The two forms have distinct pathogenic mechanisms and clinical characteristics [1,2]. Ulcerative colitis is primarily an inflammatory condition of the colonic mucosa, although deeper layers could also be involved in severe cases. UC has a characteristic diffuse distribution extending from the rectum to the proximal parts of the large bowel [3]. In the majority of patients with UC, the disease progresses with intermittent flare episodes and relapse-free remission periods. The disease activity guides the management and treatment of UC.

The main functions of the lymphatic system are fluid balance, fat absorption, and host defenses. The lymphatic system is affected in gut inflammation. Inflammatory reactions lead to increases in lymphatic flow. Impaired lymphatic drainage due to lymphatic obstruction and contractile dysfunction results in lymphangiogenesis in IBD. Lymphatic obstruction, increased lymphatic flow, and neovascularization lead to lymphatic vessel dilatation and submucosal edema [4,5,6]. Although lymphatic dysfunction presents in both forms of IBD, prominent effects of the disease on the lymphatic system are established mainly in CD [5]. 

Local inflammatory processes, including appendicitis, diverticulitis, cholecystitis, and pancreatitis cause mesenteric lymphadenopathy. In addition to local disorders, hematological malignancies, metastasis of solid malignancies, several infections, and systemic inflammatory disorders should be considered in the differential diagnosis of mesenteric lymphadenopathy [7]. Moreover, Crohn’s disease is a well-known cause of mesenteric lymphadenopathy [8]. 

Radiologic imaging has been used to define the extent and activity of IBD, in order to distinguish IBD from other diseases with the same clinical presentation, and to determine complications and extra-intestinal manifestations [9]. In UC, abdominal imaging has been used to assess the bowel wall for the extent of disease and determining activity [10,11]. There is a paucity of data regarding intraabdominal lymph nodes in UC. We aimed to investigate the presence of mesenteric lymph nodes in UC, explore lymph node characteristics according to disease activity, and compare the features of mesenteric lymph nodes that are observed in other acute intraabdominal inflammatory conditions.

## 2. Experimental Section

### 2.1. Study Population 

This retrospective study was performed in patients with UC at our center between January 2010 and May 2016. Patients were diagnosed according to clinical, radiological, and endoscopic examinations, as well as histological findings. A total of 518 consecutive patients with UC were screened, and patients who had had abdominopelvic computed tomography (CT) scanning during follow-up were recruited to the study. Data regarding clinical features, including disease duration and extension, treatments, laboratory characteristics, and disease activity during the time that abdominal CT was performed were obtained from hospital records. The disease activity was assessed by the Truelove Witts criteria and, if available, the Rachmilewitz endoscopic activity index [12,13]. Patients with UC were divided into the following two groups: patients with active disease and patients in remission. The extent of UC was determined according to proctitis, which is limited to involvement in the rectum; rectosigmoiditis, which is limited to involvement with the rectum and sigmoid colon; left-sided UC, which extends beyond the sigmoid colon up to splenic flexura; extensive colitis, which extends involvement up to splenic flexura; and pancolitis, which is involved with all parts of the colon. Patients with insufficient clinical and laboratory data and those who did not have any CT study during follow-up were excluded. Among 518 patients with UC, 100 patients were evaluated with abdominopelvic CT. Ethical approval for the study was obtained from the Institutional Review Board of Firat University Medicine Faculty (31.05.2016/10/09).

### 2.2. Radiologic Measurements and Analysis

#### 2.2.1. Multidetector Computed Tomography Imaging Protocol and Image Analysis

All CT examinations were performed using multidetector CT scanners (Toshiba Aquilion 64 slice CT, Tokyo, Japan) with 2 mm portal venous phase images (obtained 70 s after the initiation of intravenous contrast material injection). By using an automatic injector (Stellant; Medrad, Warrendale, PA, USA), 100 mL of nonionic contrast material was administered intravenously at a rate of 3–5 mL/s. The multidetector CT studies were analyzed on a picture archiving and communication system workstation (Centricity Imaging; GE Healthcare, Milwaukee, WI, USA). Lymph node attenuation was measured on axial contrast-enhanced images obtained during the portal venous phase. 

#### 2.2.2. Region of Interest (ROI) Measurements

Study radiologists (Y.E. and H.A.) measured mesenteric lymph node attenuation in all patients. For patients with multiple lymph nodes, the largest lymph node was chosen for the analysis. Lymph node attenuation was determined by placing the routinely used ROI within the lymph node’s largest dimension in the transaxial plane, and was calculated by using the measurement in a single ROI. The single ROI was drawn to cover the largest possible region within the lymph node. The measurements were made three times, and the mean value of three measurements was used for data analysis. 

### 2.3. Statistical Analysis

In this study, SPSS (Statistical Program for Social Sciences) version 22.0 for Windows was used as software. The normal distribution of the data was assessed by the Shapiro–Wilk test. Among the quantitative variables, those exhibiting a normal distribution are indicated as the mean ± standard deviation, whereas those without normal distribution are indicated as the median (25 percentile–75 percentile). Categorical variables are expressed as the number and percentage. In the two-group comparison of normally distributed quantitative variables, Student’s *t*-test was used. In two group comparisons of quantitative variables without normal distribution, the Mann–Whitney U test was used. In the comparison of categorical data, chi-square and Fisher’s exact chi-square test were used. A *p* value of less than 0.05 was accepted as statically significant.

## 3. Results

A total of 100 patients with UC (61 active and 39 inactive) were recruited. There was no difference between active and inactive UC patients in terms of age and gender (*p* > 0.05 for both). Inactive patients had longer disease duration compared to active patients (45 months versus 13 months, with *p* < 0.01). Proximal involvement was more common among active patients (52% versus 31%, with *p* = 0.03). Although the difference not reach statistically significance, immunosuppressive medications were more commonly used in active patients with UC than inactive patients (46% versus 28%, with *p* = 0.12). The clinical details of the patients with UC and controls are shown in Table 1. The CT scans had been performed concurrently with endoscopic assessments in all of the active patients with UC, and activity was defined according to the clinical signs and symptoms, laboratory features, and endoscopic findings. On the other hand, 16 inactive patients with UC had endoscopic assessments at the time of the CT scans. These patients had abdominal pain with normal stool features, and CT scans had been performed to explain the abdominal pain. The remaining 23 inactive patients had undergone CT scanning for various reasons. CT scans had been performed to explain phalanx pain, lower quadrant pain, or pelvic pain in 13 of them; to conduct external mass imaging in the rectum in two of the patients; for evaluation of cystic liver lesions in three patients; evaluation of biliary pathologies, including fascioliasis, in three patients; to exclude acute pancreatitis in one patient, presenting with abdominal pain and hyperamylasemia; and for evaluation of the portal venous system, in order to exclude portal venous thrombosis in two patients presenting with splenomegaly. Of the 23 inactive patients, the median time duration between CT scanning and endoscopic examination was 19 months (12–31 months each).

The assessment of laboratory findings and radiological lymph node (LN) measurements of the two groups are given in Table 2. The mean hemoglobin value was lower in active than in inactive UC patients (*p* < 0.01). The leucocyte counts and platelet counts were higher in active patients compared to patients in remission (*p* = 0.02 and *p* < 0.01, respectively). The erythrocyte sedimentation rates and CRP values of the active patients with UC were also significantly higher than the inactive patients (for all, *p* < 0.01). Albumin, which is a negative acute phase reactant, was found to be significantly lower in patients with active UC than inactive patients (*p* < 0.01). Other laboratory parameters including fasting glucose, blood urea nitrogen (BUN), creatinine, and alanine aminotransferase (ALT) did not differ between two groups (for all, *p* > 0.05).

The lymph node attenuation values of active UC patients and UC patients in remission were similar (49 Hounsfield units (HU) versus 58 HU, *p* = 0.31). Similarly, the mean lymph node dimension values did not differ between active patients and inactive patients (7.0 ± 1.9 mm versus 6.8 ± 2.9 mm, *p* = 0.23). According to the extent of disease (distal extension including proctitis, rectosigmoiditis and left-sided colitis, and proximal extension including extensive colitis and pancolitis), we could not detect any difference between patients who had distal extension and patients who had proximal extension in terms of lymph node attenuation (56.5 (34–67) HU versus 46 (32–60) HU respectively, *p* = 0.12) and lymph node dimension (6.7 ± 2.4 mm vs. 7.3 ± 2.1 mm respectively, *p* = 0.18). In the comparison of the groups according to the medications (mesalazine alone versus mesalazine with immunosuppressive medications), in terms of LN dimension and attenuation, only one difference was observed. The attenuation values of lymph nodes were significantly higher in the mesalazine group compared to the mesalazine and immunosuppressive combination group (59 (40–67) HU versus 41 (18–55) HU, *p* < 0.01). On the other hand, the lymph node dimensions of the mesalazine group were similar to those of the mesalazine and immunosuppressive combination group (7.1 ± 2.4 versus 6.6 ± 2.1, *p* = 0.31). In the comparison of the subgroups, according to the disease extension and activity of UC (distal inactive, distal active, proximal inactive, and proximal active) in terms of LN dimension and attenuation, only two differences were observed (Table 3). The attenuation values of the lymph nodes were significantly lower in patients with the proximally extended active and inactive disease than those in the inactive patients with distal extension (*p* = 0.04 and *p* < 0.01, respectively). 

## 4. Discussion

Mesenteric lymph nodes are associated with intraabdominal inflammation, and their presence indicates intestinal inflammation regardless of the etiology. In the current study, mesenteric lymph nodes were studied among patients with UC, which is a chronic inflammatory disorder of the large intestine.

In the gastrointestinal tract, lymphoid tissue shows variation according to the fluid clearance, fat absorption, and clearance of immune cells. Lymphoid tissue in the distal part of the ileum is more abundant than in the colonic lymphoid tissue. Chronic inflammation from IBD influences the lymphatic system, although its effect on lymphatics is correlated with the inflamed part of the gastrointestinal tract, which is more prominent in the small intestine than in the colon [5]. In normal circumstances, the lymphoid tissue of the colon is located in the muscularis mucosa layer and is absent from the rest of the mucosa. Chronic inflammation from UC results in hyperplasia of the submucosal lymphoid tissue, lymphatic angiogenesis, and lymph node formation [14,15]. Rahier et al. demonstrated the increase of mucosal lymphatic density in both inflamed and non-inflamed areas of colonic tissue in UC patients, compared with controls [16]. Lymphangiogenesis occurs in tissue and in the lymph nodes during chronic inflammation by the induction of vascular endothelial growth factors A, C, and D [17]. Alteration in the intestinal lymphatic system structure and functions is a frequent but ignored issue in IBD. Lymphangiogenesis, obstructed lymphatic vessels, lymphatic pumping dysfunction, and lymphatic obstruction results in dysfunctional lymphatics that play a crucial role in the pathogenesis and progression of IBD [18].

Data regarding lymph nodes and IBD come from investigations focused on the radiologic features of patients with CD. The lymph nodes in patients with CD are small nodes that are found adjacent to both normal and affected segments of active and inactive CD. The lymph nodes show soft-tissue attenuation and demonstrate homogeneous enhancement following intravenous contrast administration. Enlarged lymph nodes that show enhancement indicate active disease, and these nodes are detected around the vascular supply of the affected segment [8]. Despite the differences between Crohn’s disease and UC, it is believed that IBD is an inappropriate immune response to intestinal microbial antigens in genetically susceptible individuals, and lymphatic dysfunction might be a shared pathogenetic mechanism for the emergence and course of both diseases. 

To the best of our knowledge, there has been no investigation regarding the CT features of mesenteric lymph nodes in patients with UC. This retrospective study demonstrated that mesenteric lymph nodes are evident in all patients with UC, regardless of activity. 

The attenuation values of the UC patients under topical treatment (mesalazine alone) were higher than those of the UC patients using immunosuppressive medications. Lymphatic drainage of the gastrointestinal tract is a dynamic process that is regulated by several factors. One important function of lymphatics is to collect lymphocytes and interstitial fluid from the tissues and convey it for circulation. Intestinal inflammation leads to the impairment of lymphatic contractile functioning and to the accumulation of excessive interstitial fluid. These two factors result in tissue edema. Inflammation of the gut causes impairment of the clearance of immune cells, and prolonged immune cell accumulation contributes to tissue damage, especially in chronic inflammation [19]. Inflammation leads to the accumulation of interstitial fluid enriched by immune cells in the lymph nodes. The consequences of lymphatic fluid accumulation are swollen and denser lymph nodes. Our results indicate that lymph nodes have different characteristics according to the medications used, such as immunosuppressive medications. Interstitial fluid accumulation and persistent lymph stasis might be more prominent than the accumulation of immune cells under the immunosuppressive treatment. This finding could be an explanation of the low density in the lymph nodes. Ongoing systemic inflammation in the majority of the patients who are treated with only mesalazine might result in the recruitment and residence of immune system cells. The attenuation of the lymph nodes might indicate the variability of the phenotype and the course of the disease. Extension of the disease and disease activity might be important factors for lymph node characteristics. We observed that proximally extended active disease presents with lower attenuation in the lymph nodes compared with distally extended remission status. Further follow-up studies might be required with a larger numbers of cases to explore the attenuation, enlargement, and enhancement characteristics of lymph nodes in UC. 

Serum markers of inflammation have been extensively studied in UC, and the CRP increase is slight or absent; in contrast, the ESR rises less quickly but decreases more slowly, and has a lesser degree of change [20]. The ESR and CRP values were found to be higher in the active patients with UC compared with the inactive patients. The lymph node features of patients with UC were not correlated with any inflammatory marker. 

Some limitations of our study should be considered. First, the retrospective study design could be considered a limitation. Endoscopic and histopathological correlations for all patients with UC were not available. We used the Truelove Witts criteria and, if available, the Rachmilewitz endoscopic activity index to determine disease activity. Finally, the small size of the UC population might have prevented the exploration of the differences between active and inactive disease status in this analysis.

## 5. Conclusions

In conclusion, our results suggest that mesenteric lymph nodes are evident in both active and inactive forms of UC. On the other hand, disease activity and the extent of the disease do not have a clear impact on mesenteric lymph node characteristics. Further studies are needed with larger numbers of patients to explore lymph node characteristics with UC.

## Figures and Tables

**Table 1:** Clinical and demographical characteristics of active and inactive patients with ulcerative colitis (UC).

	Active UC (*n* = 61)	Inactive UC (*n* = 39)	*p*
Age (years)	47 ± 16	46 ± 13	0.56
Male, *n* (%)	40 (66)	25 (64)	0.88
Disease duration (months)	13 (4–50)	45 (30–77)	<0.01
Extent of UC, *n* (%)			0.03
Distal extension	29 (48)	27 (69)	
Proctitis/sigmoiditis	8 (13)	14 (36)	
Left sided	21 (35)	13 (33)	
Proximal extension	32 (52)	12 (31)	
Extensive	17 (28)	8 (21)	
Pancolitis	15 (24)	4 (10)	
Treatment, *n* (%)			0.12
Mesalazine alone	33 (54)	28 (72)	
Immunosuppressive *	28 (46)	11 (28)	
Corticosteroid	9 (15)	3 (8)	
Azathioprine	18 (29)	7 (18)	
Anti TNF	1 (2)	1 (2)	

* All of them used combined with mesalazine.

**Table 2:** Laboratory values and lymph node characteristics of patients with active and inactive UC.

	Active UC (*n* = 61)	Inactive UC (*n* = 39)	*p*
Hemoglobin (g/dL)	12.5 ± 2.3	14.0 ± 2.1	<0.01
Leucocyte count (/mm^3^)	8260 (6330–11,050)	6825 (5995–8995)	0.02
Platelet count (/mm^3^)	357 ± 137	281 ± 81	<0.01
ESR (mm/h)	23 (14–39)	8 (5–15)	<0.01
CRP	6.0 (3.3–16.6)	3.2 (3.1–6.0)	<0.01
Fasting glucose	94 (84–109)	88.5 (84–103)	0.42
BUN	27 ± 10	31 ± 11	0.14
Creatinine	0.76 ± 0.19	0.81 ± 0.23	0.32
ALT	21 (16–28)	22.5 (19–28)	0.28
Albumin	4.0 ± 0.6	4.4 ± 0.4	<0.01
Radiological LN characteristics			
LN dimension (mm)	7.0 ± 1.9	6.8 ± 2.9	0.23
LN attenuation (HU)	49 (37–60)	58 (31.5–68.5)	0.31

ALT: alanine aminotransferase; BUN: blood urea nitrogen; CRP: C reactive protein; ESR: erythrocyte sedimentation rate; HU: Hounsfield unit; LN: lymph node.

**Table 3:** Comparison of lymph node characteristics among the subgroups according to the disease extension and activity of UC.

	Proximal Active Disease (*n* = 32)	Proximal Inactive Disease (*n* = 12)	Distal Active Disease (*n* = 29)	Distal Inactive Disease (*n* = 27)
LN dimension (mm)	7.3 ± 1.9	7.1 ± 2.7	6.8 ± 1.8	6.6 ± 2.9
LN attenuation (HU)	47.5 (40–60)	21 (12–60.5)	54 (29–63)	59 (43.5–71)

HU: Hounsfield unit; LN: lymph node.

## References

[1] Baumgart D.C., Carding S.R. (2007). Inflammatory bowel disease: Cause and immunobiology. Lancet.

[2] Kaser A., Zeissig S., Blumberg R.S. (2010). Inflammatory bowel disease. Annu. Rev. Immunol..

[3] Ordas I., Eckmann L., Talamini M., Baumgart D.C., Sandborn W.J. (2012). Ulcerative colitis. Lancet.

[4] Von der Weid P.Y., Rainey K.J. (2010). Review article: Lymphatic system and associated adipose tissue in the development of inflammatory bowel disease. Aliment. Pharmacol. Ther..

[5] Alexander J.S., Chaitanya G.V., Grisham M.B., Boktor M. (2010). Emerging roles of lymphatics in inflammatory bowel disease. Ann. N. Y. Acad. Sci..

[6] D’Alessio S., Tacconi C., Fiocchi C., Danese S. (2013). Advances in therapeutic interventions targeting the vascular and lymphatic endothelium in inflammatory bowel disease. Curr. Opin. Gastroenterol..

[7] Lucey B.C., Stuhlfaut J.W., Soto J.A. (2005). Mesenteric lymph nodes seen at imaging: Causes and significance. Radiographics.

[8] Tolan D.J., Greenhalgh R., Zealley I.A., Halligan S., Taylor S.A. (2010). Mr enterographic manifestations of small bowel crohn disease. Radiographics.

[9] Kilcoyne A., Kaplan J.L., Gee M.S. (2016). Inflammatory bowel disease imaging: Current practice and future directions. World J. Gastroenterol..

[10] Patel B., Mottola J., Sahni V.A., Cantisani V., Ertruk M., Friedman S., Bellizzi A.M., Marcantonio A., Mortele K.J. (2012). Mdct assessment of ulcerative colitis: Radiologic analysis with clinical, endoscopic, and pathologic correlation. Abdom. Imaging.

[11] Deepak P., Bruining D.H. (2014). Radiographical evaluation of ulcerative colitis. Gastroenterol. Rep. (Oxf).

[12] Truelove S.C., Witts L.J. (1955). Cortisone in ulcerative colitis; final report on a therapeutic trial. Br. Med. J..

[13] Rachmilewitz D. (1989). Coated mesalazine (5-aminosalicylic acid) versus sulphasalazine in the treatment of active ulcerative colitis: A randomised trial. BMJ.

[14] Kaiserling E. (2001). Newly-formed lymph nodes in the submucosa in chronic inflammatory bowel disease. Lymphology.

[15] Kaiserling E., Krober S., Geleff S. (2003). Lymphatic vessels in the colonic mucosa in ulcerative colitis. Lymphology.

[16] Rahier J.F., De Beauce S., Dubuquoy L., Erdual E., Colombel J.F., Jouret-Mourin A., Geboes K., Desreumaux P. (2011). Increased lymphatic vessel density and lymphangiogenesis in inflammatory bowel disease. Aliment. Pharmacol. Ther..

[17] Liao S., von der Weid P.Y. (2015). Lymphatic system: An active pathway for immune protection. Semin. Cell Dev. Biol..

[18] Ge Y., Li Y., Gong J., Zhu W. (2018). Mesenteric organ lymphatics and inflammatory bowel disease. Ann. Anat..

[19] Alexander J.S., Ganta V.C., Jordan P.A., Witte M.H. (2010). Gastrointestinal lymphatics in health and disease. Pathophysiology.

[20] Cioffi M., Rosa A.D., Serao R., Picone I., Vietri M.T. (2015). Laboratory markers in ulcerative colitis: Current insights and future advances. World J. Gastrointest. Pathophysiol..

